# Automated cell segmentation in FIJI® using the DRAQ5 nuclear dye

**DOI:** 10.1186/s12859-019-2602-2

**Published:** 2019-01-18

**Authors:** Mischa Schwendy, Ronald E. Unger, Mischa Bonn, Sapun H. Parekh

**Affiliations:** 1 Max Planck Institute for Polymer Research, Ackermannweg 10, 55128 Mainz, Germany; 2 Institute of Pathology, Universitätsmedizin-Mainz, Langenbeckstraße 1, 55131 Mainz, Germany

**Keywords:** Cell segmentation, Image processing, Batch processing, Fiji, ImageJ, DRAQ5

## Abstract

**Background:**

Image segmentation and quantification are essential steps in quantitative cellular analysis. In this work, we present a fast, customizable, and unsupervised cell segmentation method that is based solely on Fiji (is just ImageJ)®, one of the most commonly used open-source software packages for microscopy analysis. In our method, the “leaky” fluorescence from the DNA stain DRAQ5 is used for automated nucleus detection and 2D cell segmentation.

**Results:**

Based on an evaluation with HeLa cells compared to human counting, our algorithm reached accuracy levels above 92% and sensitivity levels of 94%. 86% of the evaluated cells were segmented correctly, and the average intersection over union score of detected segmentation frames to manually segmented cells was above 0.83. Using this approach, we quantified changes in the projected cell area, circularity, and aspect ratio of THP-1 cells differentiating from monocytes to macrophages, observing significant cell growth and a transition from circular to elongated form. In a second application, we quantified changes in the projected cell area of CHO cells upon lowering the incubation temperature, a common stimulus to increase protein production in biotechnology applications, and found a stark decrease in cell area.

**Conclusions:**

Our method is straightforward and easily applicable using our staining protocol. We believe this method will help other non-image processing specialists use microscopy for quantitative image analysis.

**Electronic supplementary material:**

The online version of this article (10.1186/s12859-019-2602-2) contains supplementary material, which is available to authorized users.

## Background

Fluorescence microscopy is the method of choice to visualize specific cellular organelles, proteins, or nucleic acids with high sensitivity and selectivity. Importantly, fluorescence is, in principle, quantitative in that intensity of fluorescence from each position in a sample is proportional to the abundance of the fluorescent moiety in that region of the sample. Once fluorescence images are properly corrected, quantitative image processing can provide abundant information about the imaged species – most notably its spatial distribution within single cells [1–3]. The commercialization of automated microscopes, together with thousands of different fluorescent proteins, cell stains, and digital microscopy, has catalyzed the production of a staggering amount of high-quality imaging data. Thus, it is indispensable to automate the process of image quantification of which one essential step is image segmentation, i.e., the selection and compartmentalization of regions of interest (ROI) within the image. In mammalian cell culture experiments, which are the focus of this work, these ROIs are quite often single cells.

Proprietary image processing software from microscope manufacturers or software specialists such as Imaris or Metamorph offer potent and ready-to-use solutions for image segmentation and further processing. These programs are user-friendly and do not require deep knowledge of data processing nor any programming skills but require a monetary expenditure. CellProfiler is an open-source, alternative tool that offers a platform with a graphical user interface to customize a pipeline for cell detection and geometric quantification based on pre-programmed methods [2]. The method presented in this work is an algorithm built within FIJI (is just ImageJ)® – hereafter called FIJI, a popular and effective alternative to CellProfiler, which is bundled with the open-source Micro-Manger microscopy control software [4, 5]. Because FIJI is widely used in the microscopy community, it offers a broad toolbox with several basic and (user-provided) advanced processing steps (via plugins) that can be combined to produce powerful image processing methods.

Automated fluorescence microscopy based cell segmentation algorithms from cytoplasmic stains can exhibit correct segmentation results above 89% [6]. Modern computer vision algorithms for cell microscopy generate highly accurate segmentation lines with intersection over union (IoU) scores above 0.9, even for unstained samples (U-Net) [7]. However, training computer vision algorithms requires large annotated datasets and can be challenging to adapt for additional imaging modalities when the training dataset does not sufficiently account for image diversity. In this contribution, we present a practical, automated algorithm for mammalian cell segmentation and geometric feature quantification in FIJI that can be extracted from fluorescent images using a single nuclear stain – in this case, DRAQ5, as opposed to more frequently used cell body stains. Because DRAQ5 does not exhibit fluorescence enhancement upon intercalating into DNA, as opposed to the almost omnipresent DAPI, it produces a moderate, “leaky”, cytosolic fluorescent DRAQ5 signal, which is still detectable within the dynamic range of our PMT in the confocal microscope. This “leaky” signal is crucial for our cell segmentation method. Our algorithm is based on appropriate background subtraction and the identification of the weak cytosolic DRAQ5 signals to properly identify cell bodies. Subsequent watershedding using the strong nuclear signal as the respective local maxima allows for efficient, and more importantly, accurate cell border detection. The modularity and delivery of our algorithm as an ImageJ macro should make it readily customizable to other end user’s needs. Moreover, it should be no problem to use this algorithm with other nuclear dyes so long as the dye exhibits sufficient cytosolic fluorescence along with strong nuclear fluorescence.

We start by describing the algorithm and demonstrating its quantitative accuracy by comparing automated analysis against human detection of HeLa cells. In two applications of our algorithm, we analyzed the cell growth of THP-1 cells during differentiation and the change in spreading area of Chinese hamster ovary (CHO) cells during low-temperature cultivation – a perturbation regularly used for biotechnology applications [8].

## Results

### Algorithm development

**Scheme 1:**
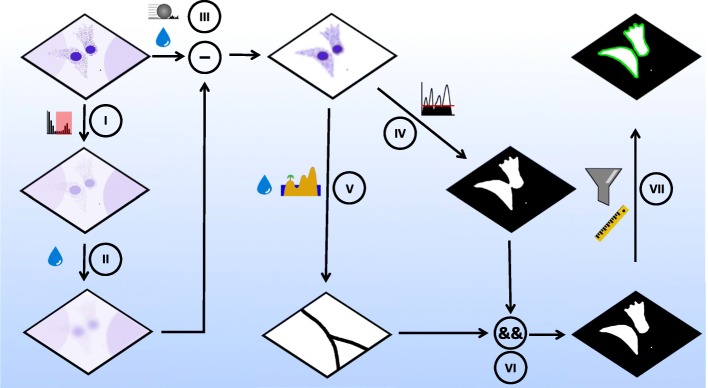
Steps in image processing and segmentation algorithm. (I,II): Production of a subtraction mask for background subtraction by duplicating the raw image and constraining maximum to three-fold the mean gray value of the image (I). Gaussian blurring (shown here as the water droplet) of the constrained image (II) generates a background image for background subtraction in III. III: The background in the original Z-projected image is reduced via a double subtraction step. First, a rolling ball subtraction is performed (ball radius is set larger than cell radius, to leave the cytosolic signal unaffected) with a subsequent Gaussian blurring of remaining punctate background signals. Secondly, the background image (made in II) is subtracted from the (already background reduced) version of the original image. This background subtraction procedure results in an almost flat background image containing only nuclear and cytosolic intensity components. IV: Thresholding the blurred, background-subtracted image results in a binary cytosol mask. V: The image from III was further blurred, and watershedded to produce a binary image of lines that split touching cells. VI: The logical (pixel-wise) AND operation combined cytosolic mask (from V) and the watershed lines (from VI) to a binary image of segmented cells. VII: Particle analysis with a size filter allows neglecting small particles in the image and selection of the segmented cells for further analysis

The overall processing scheme is outlined below in Scheme 1. All image processing was performed on a Z-projected image – projected according to the standard deviation – of DRAQ5 fluorescence; each pixel in the projected image had an intensity value given by the standard deviation of the pixels in Z-direction. This highlights zones with a high degree of variation in the Z-direction, which enhances weak and punctate signals. To reduce the background signal in Z-projected images caused by uneven illumination or non-specifically bound fluorophores, we applied a two-step process. First, a background image was produced by constraining the maximum of the original image and Gaussian blurring the constrained image with a sigma of 100 μm (Scheme 1**, I and II**). This process coerced high-intensity signals to a new maximum value of the threefold global mean gray value and blurred the leaky cytosolic DRAQ5 signal so that it would remain after subsequent subtraction from the original image. This background image was subtracted from the pre-flattened version of the original Z-projected image that was generated using a rolling ball subtraction with a radius of 100 μm followed by smoothing with a Gaussian blur filter with a sigma of 1 μm. Subtracting the background image from the processed original image resulted in a near-zero background except for the nuclear and cytosolic DRAQ5 signals (Scheme 1**, III**). The resulting image (from step III) was duplicated and used for thresholding and watershedding (Scheme 1**, IV and V**). Specifically, to produce a binary image of the cell bodies, a threshold at a gray value of 1 was sufficient as all background values were strongly reduced (Scheme 1**, III**). This produced a binary image that highlights complete cell bodies and nuclei. Adjacent and overlapping cells were divided in the further Gaussian blurred (sigma = 2 μm) copy from step III by applying the “find maxima” command with “Segmented Particles” as the output and a noise value given by a threefold mean gray value of all non-zero gray values in the image (Scheme 1**, V**).

Combining the thresholded image (Scheme 1**, IV**) with the watershed lines (Scheme 1**, V)** via the logical (pixel-wise) “AND” operation produced a binary mask of the cell population in the image with juxtaposed cell borders of individual cells separated (Scheme 1**, VI**). A size filter was applied, detecting cells with sizes bigger than 200 μm^2^ to avoid detection of cell debris (Scheme 1**, VII**).

To analyze cell shape, we probed three parameters: projected cell area as a measure of cell spreading, circularity as a measure of cellular protrusions and aspect ratio as a measure of elongation. These quantities are exported in an automated fashion in a table format at the end of the analysis. An example of the Macro is given in the Additional file 1. All images used in this work and the example Macro is additionally available in [9].

### Evaluation of the cell detection and segmentation method

**Fig. 1:**
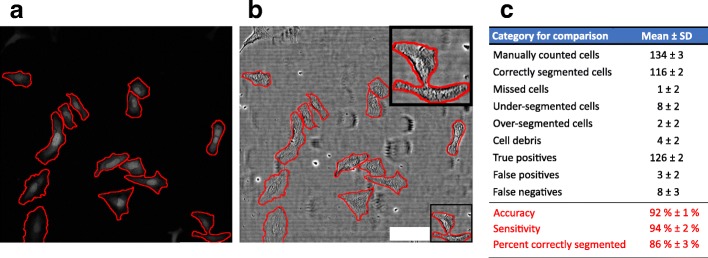
Exemplary output of cellular detection from leaky DRAQ5 staining and evaluation of the automated segmentation algorithm. **a** Z-projected DRAQ5 signal. **b** Z-projected brightfield (transmitted laser light) image. Inset shows an example of undersegmented cells. Red lines in a and b are segmentation lines produced by our algorithm. **c** Quantitative evaluation of the segmentation algorithm showed mean accuracy, sensitivity, and correct segmentation values of 92, 94, and 86%, respectively, when compared to human detections (from three individuals, each with more than three years of experience in cell culture). The specific categories are defined in the Methods section. The evaluation was performed on 136 cells in *n* = 8 images from two experiments. Scale bar is 100 μm

An exemplary output of segmented cells within a processed image is shown in Fig. 1a. To evaluate the segmentation from the leaky DRAQ5 signal, we used an established approach that relies on manually monitoring the automated detection results on a set of test images [10]; similar human-comparison approaches have been used elsewhere [11, 12]. The evaluation was performed by applying the selected frames on the corresponding bright-field image (Fig. 1b), and three individuals, each with more than three years of experience in cell culture, manually counted cells and checked for appropriate segmentation produced by our automated algorithm. Manually checking for correctly segmented cells, true positive (including full cell bodies, largest fragment of over-segmented cells and one cell per under-segmented multi-cell detection), false positive (cell debris, thresholding errors, etc.) and false negative (missed cells, undetected cells in under-segmented multi-cell detections) detections from our algorithm, we found that 86% of cells were correctly segmented, with accuracy and sensitivity values better than 92% (Fig. 1c**)**. Additionally, comparing the overlap of manually segmented cells (as the ground truth) with algorithm detections yielded an intersection over union (IoU) score (explained in the Methods) of 0.83 ± 0.05.

Having established that our segmentation algorithm was accurate and specific compared to human evaluation and with respect to IoU scores compared to literature (see Discussion for details), we next focused on demonstrating the application of this method in different cell biology applications. We quantifed geometrical features of THP-1 cells during differentiation and spreading characteristics of CHO cells during low-temperature cultivation (often used to increase protein production yield in biotechnology applications – reviewed in [8]).

### Quantifying projected area, aspect ratio, and circularity of HeLa and THP-1 from automated cell segmentation

**Fig. 2:**
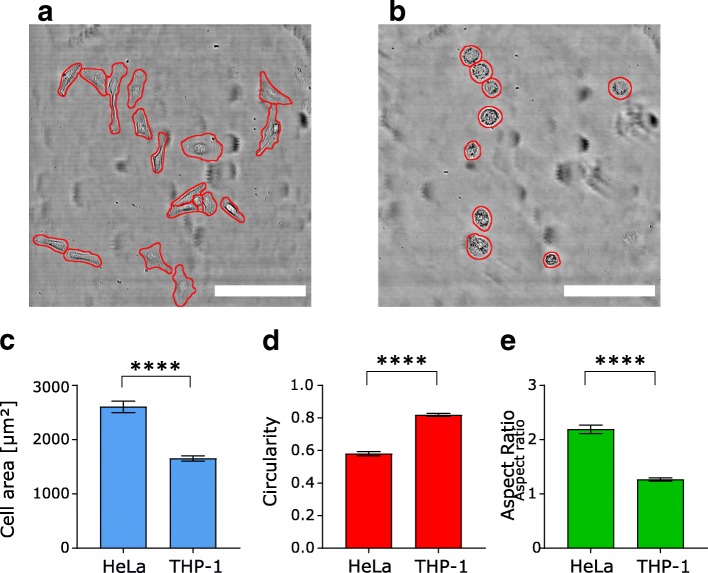
Exemplary images and shape descriptors for HeLa and differentiated THP-1 cells. **a** Brightfield (transmitted laser light) image of HeLa with segmentation lines (red) produced by our algorithm. **b** Brightfield (transmitted laser light) image of differentiated THP-1 with segmentation lines (red) produced by our algorithm. **c** Mean projected cell area of differentiated THP-1 and HeLa cells shows that THP-1 cells have a 36% smaller area compared to HeLa. **d** Quantification of cell circularity confirms that THP-1 cells have a 41% higher (mean) circularity value compared to HeLa. **e** Elongated HeLa cells show a mean aspect ratio above 2 while the round shape of THP-1 is reflected by an aspect ratio ~ 1. Data are shown as mean ± standard error of mean (sem). **** indicates *p* < 0.0001 (t-test). For HeLa, 129 cells in n = 8 images from two pooled experiments (the same images as in Fig. 1) were analyzed; for THP-1, 135 cells in *n* = 13 images from two pooled experiments were analyzed. Scale bar is 200 μm in both images

To demonstrate the ability of our algorithm to measure cell morphology accurately, we compared two cell lines with different growth behavior: HeLa and THP-1 cells. We picked freshly differentiated THP-1 (after 48 h differentiation and 24 h of recovery) showing a predominantly round, almost protrusion-free shape and HeLa cells showing a larger, more elongated cell shape (Fig. 2a and b). Both cell lines were cultured on collagen-coated glass-bottom MatTek dishes (as supplied by the manufacturer).

To quantify the different shape characteristics, we measured the projected cell area, cell aspect ratio, and circularity. Briefly, the aspect ratio is defined as the ratio of the major to the minor axis of a fitted ellipse; circularity is defined as 4*(area/perimeter^2^). For example, objects having the shape of a perfect circle, have circularities and aspect ratios equal to 1. Higher aspect ratios are associated with elongation; lower circularity values are associated with cellular protrusions. Detailed information on these parameters is given in the Additional file 1 (Additional file 1 Figure S1).

We found that HeLa cells show a projected cell area of ~ 2600 μm^2^, a mean circularity of 0.58 and a mean aspect ratio of 2.2. In contrast, differentiated THP-1 showed a cell area of ~ 1650 μm^2^ and were almost perfectly round with a mean circularity of 0.82 and a mean aspect ratio of 1.27 (Fig. 2c - e). These findings are consistent with the elongated form of HeLa and the smaller round shape of differentiated THP-1 seen in the images of Fig. 2a and b, respectively.

### Cell shape changes during THP-1 differentiation

Previous work has shown that THP-1 cells change their phenotype dramatically during differentiation, as they undergo a transition from suspension to adherent cells during differentiation into macrophage-like cells [13, 14]. Therefore, we analyzed cell morphology changes by monitoring cell area, circularity, and aspect ratio from dozens of confocal stacks that contained more than 90 (per day) THP-1 cells over a six-day differentiation and culture period.

**Fig. 3:**
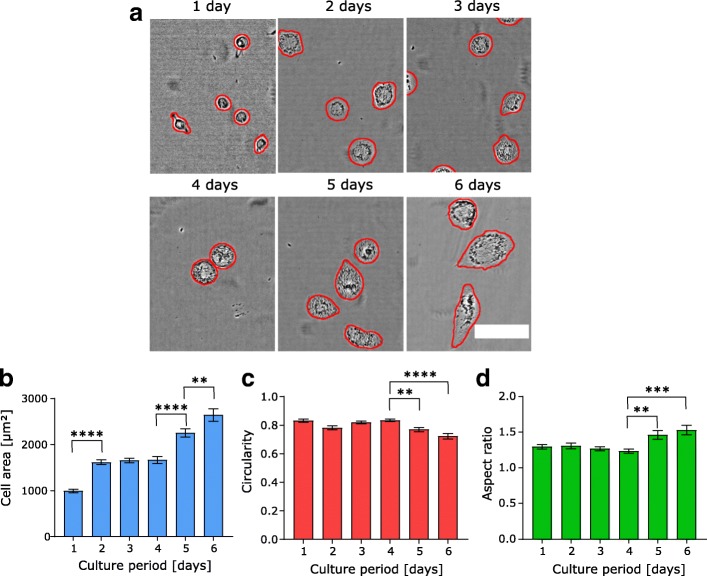
Change in cell area, circularity and aspect ratio during THP-1 differentiation. **a** Exemplary brightfield (transmitted laser light) images of THP-1 cells during the 6 day monitoring phase with segmentation lines (red) produced by our algorithm. **b** Projected cell area increases during differentiation. After an initial growth phase, cells enter a plateau phase and then show a second period of growth after day 4. **c** Circularity values stay constant until day 4 before decreasing on day 5 and 6. **d** Increasing aspect ratio on day 5 and day 6 indicates cellular elongation. These data together highlight the shape change toward a more elongated phenotype at longer times after differentiation. Data are shown as mean ± sem. **,*** and **** indicate *p* < 0.01, 0.001 and 0.0001, respectively (ANOVA with post-hoc Tukey test). The number of cells for each day, starting from day 1 to day 6: 110, 114, 135, 113, 121, 91 with at least *n* ≥ 10 images were analyzed from two pooled experiments per day. Scale bar is 100 μm

Incubating THP-1 with PMA in the culture medium for 48 h (to initiate differentiation) and subsequently changing to normal medium without PMA resulted in the spreading phenotype shown in Fig. 3a. Cell area increased sharply within the first 48 h, subsequently entering a plateau phase without further growth for the next 48 h before the cells entered another growth phase after day 4 (Fig. 3b). Interestingly, this second growth phase appears to be accompanied by further shape changes, as both circularity and aspect ratio show statistically significant changes only after day 4. The decreased circularity along with the increased aspect ratio indicates cellular elongation and less smooth, round shapes (Fig. 3b and c), consistent with the shapes seen in Fig. 3a.

### Reduced temperature culture of CHO-cells causes cell shrinkage

As a second application of our cell segmentation algorithm, we analyzed the impact of temperature on projected cell area of CHO cells. Numerous studies have demonstrated how reduced temperature affects cellular growth (via arrest) and increases protein production in CHO cells [15, 16]. Higher rates of recombinant protein expression, coupled with extended production phases make temperature an interesting and easily tunable parameter in industrial biotechnological upstream processing.

**Fig. 4:**
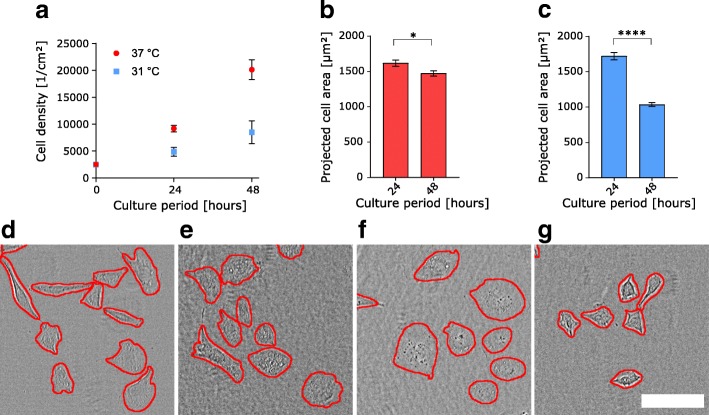
Cell area changes during low-temperature culture of CHO cells. **a** Cell surface density and growth is reduced by 48 h of CHO culture at 31 °C compared to 37 °C. *n* = 3 experiments per temperature and day. **b**,**c** Projected cell area of CHO cells cultured at 37 °C and 31 °C, respectively, shows that culture at 31 °C culture results in a sharp decrease in cell area after 48 h. For (**b**) *n* = 218 cells at 24 h and *n* = 494 cells at 48 h in 10 images from 2 pooled experiments, respectively. For (**c**) *n* = 217 cells at 24 h and *n* = 461 cells at 48 h in 10 images from 2 pooled experiments, respectively. (**d** – **g**) Exemplary brightfield (transmitted laser light) images of CHO cells with segmentation lines (red) produced by our algorithm cultured at 37 °C for 24 h (**d**), 37 °C for 48 h (**e**), 31 °C for 24 h (**f**), 31 °C for 48 h (**g**). All graphical data are shown as mean ± sem. * and **** indicate *p* < 0.05 and 0.0001, respectively (t-test). Scale bar is 100 μm

Quantifying cell densities per cm^2^ we found, similarly to prior studies, that CHO cells cultured after a temperature reduction to 31 °C show decreased cell growth [17]. We detected half the growth rate seen for culture at 31 °C compared to control conditions (37 °C) (Fig. 4a). To evaluate whether this temperature jump had an impact on cell shape, we analyzed the projected cell area of adherent CHO cells cultured at either 31 °C or 37 °C temperature over 48 h. As shown in Fig. 4b, cell spreading decreased by less than 10% after 48 h of culture at 37 °C (that is, with no temperature perturbation). On the other hand, for CHO cells cultured at 31 °C, we observed values comparable to control conditions after 24 h of culture, but a steep drop of 40% in projected cell area after 48 h (Fig. 4c). This area reduction can also be seen in the exemplary images in Fig. 4d – g.

## Discussion

In this study, we showed that it is possible to produce robust and accurate cell segmentation algorithms in FIJI with high accuracy and sensitivity using only a leaky signal from a nuclear stain. Because no additional cell body stain was necessary, this method frees a color for additional cell staining. Our algorithm produced better than 92% accuracy, 94% sensitivity, and 86% correctly segmented cells compared to human evaluation. This places our algorithm in similar segmentation performance as reported by Wählby et al. (above 89% correct segmentations) [6] and Buggenthin et al. (accuracies above 82% and sensitivities above 94%) [10]. Additionally, we achieved an average IoU score of 0.83 when comparing our segmentation results to manual segmentation masks. This matches IoU scores of modern computer vision applications reported by Ronneberger et al. (0.77–0.92) [7].

We suspect that characteristics leading to incorrect counting from our algorithm include substantial cell clumping and a significant contribution from 3D cell growth. In these cases, several nuclei overlay, which ultimately hinders the watershedding process. For optimal quantitative analysis with our method, cells should not exhibit excessive clumping and should be preferably maintained in 2D culture. Additionally, fully confluent cell layers can be a hindrance, as they amplify the values in the subtraction mask and thereby lead to a reduction of “leaky” cytosolic signal in step III of the algorithm.

Compared to other algorithms that perform similar functions – automated segmentation and cell quantification, our algorithm offers both functions while using only a single nuclear dye that can also be used for binary cell counting, is conceptually straightforward, and built on an open-source (FIJI) platform. The comparison in terms of accuracy is on par with other approaches [18, 19] in terms of the specificity and error rate. Moreover, the general approach of thresholding combined with edge-detection to outline full cell bodies is in line with classical methods used for microscopy image segmentation [1]. Similar methods have recently been used to evaluate bacterial segmentation [20–22] in the processing suites called MicrobeTracker, CellShape, and SuperSegger, respectively. Of these, MicrobeTracker was recently translated into a FIJI plugin called MicrobeJ [23].

### The projected area of HeLa and differentiated THP-1 cells

Determination of cell area of HeLa cells using our algorithm resulted in larger cell areas than reported in literature. However, this can be traced back to different cell culture conditions: Puck et al. measured the cell area (1600 μm^2^) in 1956 using a self-made medium, and it is unclear if this contains similar supplements and additives as is common in today’s RPMI-based medium [24]. Missirlis reported a cell area of 1400 μm^2^ for HeLas cultured on a “stiff” substrate, which was a hydrogel of ~ 85 kPa [25]. As we examined cell area on glass with a Young’s modulus of order of GPa and because adherent cells tend to increase area with increasing stiffness [26], a bigger cell area in our experiments is not surprising. Lastly, Frank et al. reported a majority of HeLa cells analyzed show areas below 1100 μm^**2**^ [27]. However, these measurements were performed only one hour after cell plating, which may still be during the initial spreading phase and is not comparable to our 24 h culture period. Taken together, and considering the accuracy of the cell outlines shown in our segmentation method, we surmise that our experiments give an accurate quantification of HeLa cell area under standard laboratory conditions after 2 days of seeding on glass bottom dishes.

### Cell shape changes during THP-1 differentiation

Our results showed two growth periods of THP-1 from day 1 to 2 and from day 4 onwards, divided by a plateau phase with only minimal growth from day 2 to 4. Simultaneously, after day 4, we observed a trend to less circular, more elongated cell shape. This indicates the tendency to more pronounced cell spreading after a recovery phase of two days following PMA withdrawal. Our findings also potentially raise the question for several studies performed with THP-1 as to whether cell area was taken into consideration, as the increased membrane surface can influence cellular uptake of nutrients and the total number of membrane receptors. Many parameters such as cytokine expression, volume, and lysosomal numbers in THP-1 have been analyzed in detail [14]; however, to the best of our knowledge projected cell area during differentiation has not been correlated to these properties. It might be of further interest to analyze, e.g. cytokine excretion or lipid uptake – processes critical in immunology and pathogenesis – as a function of cell area to see how these parameters are linked to cell shape.

### Changes in projected cell area with low-temperature culture

We found a 1.7-fold decreased projected area of CHO cells after 48 h of culture at 31 °C. Interestingly, Kaufmann et al. reported a 1.7-fold increased specific protein productivity in CHO after lowering the culture temperature from 37 °C to 30 °C [17]. These higher production rates may be attributed to CHO cells adopting a quiescent reproduction phenotype, with fewer cell divisions and an accompanying smaller cell area. This could potentially free metabolic resources that could be directed toward protein production; however, this question certainly requires further exploration.

## Conclusions

In this work, we demonstrated an automated process for mammalian cell image segmentation within the open-source scientific image analysis platform FIJI. Our method was developed to segment and identify cells from Z-projected images of the DRAQ5 nuclear dye and produced accuracy levels above 92%, sensitivity levels of 94, and 86% correctly segmented cells when compared to human evaluation. Using the precise IoU metric, our segmentation gave an IoU score of 0.83; all metrics which are very close to other published algorithms. Applying our algorithm, we measured cell spreading and elongation during THP-1 differentiation to macrophages and cell area reduction of CHO cells that arises in low-temperature cultures often used for protein production. At present, the majority of cell segmentation algorithms, including ours, are based on hard-coded detection of fluorescently-labeled image species. However, with emerging algorithms, especially in the field of computer vision and deep learning, future cell segmentation and analysis could transition to label-free (e.g. brightfield) imaging that enables unperturbed, label-free, and robust monitoring of cell shape as has already been demonstrated for phase contrast and differential interference contrast imaging [7, 28, 29].

## Methods

### Cell culture and staining

Unless stated otherwise, all cell culture experiments were performed at 37 °C and 90% relative humidity, with 10% fetal calf serum (Gibco) and 10 U/mL Penicillin/Streptomycin (Gibco) added to the respective medium, and with glass bottom culture vessels (MatTek).

HeLa cells (DSMZ no: ACC 57) were cultured in DMEM (Gibco) and THP-1 cells (DSMZ no: ACC 16) in RPMI 1640 medium (Sigma). To initiate differentiation, THP-1 culture medium was supplemented with 100 ng/mL phorbol 12-myristate-13 acetate (PMA) for 48 h as previously described [30]. This procedure led to the adhesion of almost all cells within 24 h, which signals the onset of differentiation from monocytes to macrophages [14]. For analysis of temporal changes in cell area during differentiation, THP-1 cells were incubated for an additional 96 h in full medium without PMA.

Chinese hamster ovary (CHO) cells (CHO-K1, DSMZ: ACC110) were cultured in Ham’s F12 medium. For cell density determination, CHOs were cultured in 6-well-plates (Greiner Bio-One) and counted in triplicate using a hemocytometer. For cell area determination, CHO cells were cultured in glass bottom μ-dishes (Ibidi).

After the indicated incubation times, all cells were fixed with 4% para-formaldehyde in PBS for 10 min. Cells were stained with 5 μM DRAQ5 (ThermoFisher) in phosphate-buffered saline (PBS) for 40 min at 37 °C and washed with PBS three times prior to microscopic analysis. Microscope measurements were performed within 24 h for all experiments.

### Image acquisition

Confocal microscopy (Leica TCS SP5 II, Leica) of cells was used to acquire axial cell volumes. More than 100 individual cells of each cell line were imaged using a 25X, 0.95 NA water immersion objective (Leica) with a 632.8 nm HeNe laser excitation. Emission was detected from 680 to 730 nm. Detector gain was set to minimize saturation within the nucleus, and the slice thickness within the Z-stack was set to 1.51 μm. The X-Y spacing was set to 0.6 μm per pixel, and the scanner speed was 400 Hz.

### Algorithm evaluation

Three individuals, each having more than three years of cell culture experience, manually evaluated the segmentation results on a test image set. The following paragraph summarizes the measured observables, similarly defined by Buggenthin et al. [10].

“Manually counted cells” denotes all cells that are completely contained in the image. Cells found by the algorithm that have more than 90% of their area within the detected cell frame were counted in the category “correctly segmented cells”. “Missed cells” are cells in the image that were not detected by the algorithm. To account for segmentation quality, the two categories “under-segmented cells” and “over-segmented cells” were included. “Under-segmented cells” are multiple cells that are detected as a single instance such that the detected frame contains more than one cell or single cells that are detected by the algorithm where the frame is much larger than the actual cell. “Over-segmented cells” are instances where only a small section of the cell is detected, or one cell is split into multiple parts. Large cell debris in the image that could potentially be mistaken as a cell by a segmentation algorithm were counted in the “debris” category. For calculation of “accuracy” and “sensitivity”, detected instances were categorized as “true positives” (cells correctly identified by the algorithm, no information about segmentation), “false positives” (cell debris or any other objects that were falsely detected by the algorithm), and “false negatives” (any cells that were not detected by the algorithm in addition to those that were not counted in under-segmented instances). Accuracy was calculated as 100*(true positives)/(true positives + false positives + false negatives) and sensitivity as 100*(true positives)/(true positives + false negatives). “Percent correctly segmented” was calculated as 100*(correctly segmented cells) / (manually counted cells).

Additionally, to quantify the segmentation success, binary ground truth masks of cells in all test images were produced manually, and the intersection over union (IoU) score was calculated for the algorithm segmentation results using the FIJI plugin MorphoLibJ [31].

### Statistical analysis

Statistical evaluation was performed using GraphPad Prism 7.0 (GraphPad Software). To test the statistical significance of differences in the cell area, circularity, and aspect ratio of THP-1 and HeLa cells, as well as cell area differences of CHO cells cultured at different temperatures, a two-tailed t-test with Welch’s correction, was applied. For time-dependent changes in cell area, circularity, and aspect ratio during THP-1 differentiation, data were evaluated using analysis of variance (ANOVA) with post-hoc analysis based on the Tukey test. Statistical significance was expressed as *, **,*** and ****, indicating *p*-values < 0.05, 0.01, 0.001 and 0.0001, respectively.

## Additional file

Additional file 1: Additional information, methods, and macro code. (DOCX 57 kb)

## Abbreviations

ANOVA: Analysis of variance
PMA: Phorbol 12-myristate-13 acetate
