# Supplementary material for: Automated cell segmentation in FIJI® using the DRAQ5 nuclear dye
Source: BMC Bioinformatics. 2019 Jan 18;20:39. doi: 10.1186/s12859-019-2602-2 (PMC6339324; doi:10.1186/s12859-019-2602-2)
Supplement: Supplementary file 1 — Additional information, methods, and macro code. (DOCX 57 kb) [file 12859_2019_2602_MOESM1_ESM.docx]

# Additional file 1

**Automated cell segmentation in FIJI® using the DRAQ5 nuclear dye**

Mischa Schwendy^1,*^, Ronald E. Unger^2^, Mischa Bonn^1^, Sapun H. Parekh^1,*^

^1^ Max Planck Institute for Polymer Research, Ackermannweg 10, 55128 Mainz, Germany

^2^ Institute of Pathology, Universitätsmedizin-Mainz, Langenbeckstraße 1, 55131 Mainz, Germany

^*^ corresponding authors: schwendym@mpip-mainz.mpg.de, parekh@mpip-mainz.mpg.de

**Geometric features**

**Circularity – a measure of small cell protrusions**

The circularity parameter relates the area of a detected object to its perimeter.

$$Circ=4 \cdot\frac{Area}{{(Perimeter)}^{2}}$$

If the object has a perfectly circular shape, i.e., possesses the largest possible ratio of area to perimeter, the circularity parameter equals 1. From that state, it is possible to create an object with an increased perimeter while its area stays constant, but not vice versa. Thus, it follows that circularity will approach 0 with increasing perimeter in the denominator.

A cell will have lower circularity values when its silhouette is more variegated (see **Figure S1**). Lower circularity can be observed with several phenotypes including one-dimensional elongation, the formation of pseudopodia or with the apparent surface roughness formed by lamellipodiae. Therefore, one should not rely on circularity as a single descriptor.

**Aspect ratio – a measure for cell elongation**

If the goal in an experiment is to probe the elongation of cells, the best marker is the aspect ratio as it relates the length of the major axis to the length of the minor axis of an ellipse that is fitted to the object. High values in this parameter are directly linked to a high degree of elongation.

$$AR=\frac{major axis}{minor axis}$$

Unfortunately, pitfalls arise for curved cells or cells with multiple protrusions.


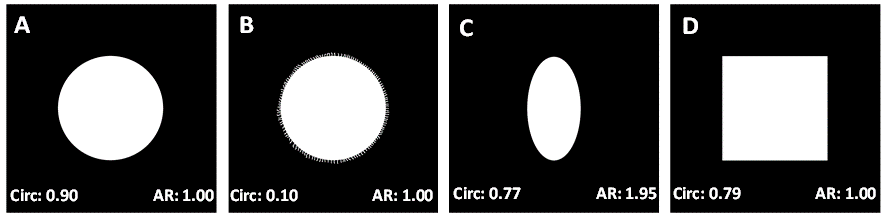


**Figure S1.** Circularity (Circ) and aspect ratio (AR) of different geometric objects; **a)** perfect circle with a circularity of 0.90 (in theory 1.00, but decreased because pixelation creates a finite amount of surface roughness around a curve) and aspect ratio of 1.00. **b)** circle with high surface roughness, and an extended perimeter leading to a circularity of 0.10, but leaving AR unchanged. **c)** Elongated ellipse, with circularity of 0.77 and an aspect ratio of 1.95. **d)** A square object with circularity of 0.79 and aspect ratio of 1, because the ellipse fit leads to the assumption of a circle.

Taken together, along with the projected cell area and perimeter, these quantities provide a basic toolbox for quantitative cell shape description.

**Image processing steps applied in the published algorithm**

Our cell detection and segmentation algorithm makes use of several built-in FIJI processing functions that are briefly explained below.

1. Rolling ball background subtraction is background reduction process. It assumes that a grayscale image is a 3D surface with the intensity values corresponding to the topography. A ball “rolls” over this landscape, averaging the values within its radius and subtracting the average value from the raw value of the central pixel in the ball in original image.
2. Gaussian blurring smoothing method. A Gaussian kernel with the width is convolved with the image intensities in the raw image to obtain a smoothed image. By convolution, one obtains a spatially smoothed image with Gaussian weighted contributions from neighboring pixels in the original image.
3. Intensity coercion via a constrained maximum sets a ceiling for the maximum intensities in an image.
4. The watershed algorithm contained in the “Find maxima…” function with output “Segmented Particles” is a tool to separate touching objects, in this case on grayscale images. It functions by considering the intensities as topography where valleys (or basins) are flooded with water to detect “watershed” lines between the hills. These lines correspond to the regions where two objects are touching, thereby offering the possibility to split them into individual objects. Importantly, the noise value must be chosen appropriately, in this case below the values for local maxima, to avoid over-segmentation as explained previously by Meyer and Beucher [1].

References

1. Meyer F, Beucher S: **Morphological segmentation**. *Journal of Visual Communication and Image Representation* 1990, **1**(1):21-46.

**Macro code**

//settings

//This code works best with Leica image files (.lif); adjust code for other file formats

f = "[Cell_summaries]"; //title of the produced

x = 1; //nucleus channel

y = 2; //brightfield channel

input = "C:\\path\\to\\input\\file.lif"; //input file

output = "C:\\path\\to\\output\\directory\\"; // output directory

cellradius = 100; //maximum expected cell radius in µm

seriesnum = 13; //Number of series in the leica image file

//Start of procedure

for (j = 1; j <= seriesnum; j++){

run("Close All"); //previously opened images are closed to avoid mislead processing of old images with same names

//Create table to save results

run("New... ", "name="+f+" type=Table");

print(f,"\\Headings:Cell\tarea\tCirc\tAR\tRoundness\tMaximum");

//Importing images using Bio-Formats Importer

run("Bio-Formats Importer", "open="+input+" autoscale color_mode=Default split_channels view=[Standard ImageJ] stack_order=Default series_"+j+"");

name = getTitle();

prefix = substring (name, 0, lastIndexOf(name," "));

run("Clear Results");

//producing duplicates

selectWindow(""+prefix+" C="+x+"");//Nucleus channel

run("Z Project...", "projection=[Standard Deviation]");//picture for frame detection

run("8-bit");

run("Duplicate...", "title=IMAGE");//frame

run("Duplicate...", "title=SUBTRACT");//Background subtraction mask (for frame and watershed)

getPixelSize(unit, pixelWidth, pixelHeight);

//create subtraction mask, applying constraining maximum (step I)

selectWindow("SUBTRACT");

getVoxelSize(w, h, d, unit);

getStatistics(area, mean, min, max, std);

row = nResults;

setResult("Max ", row, max);

u=floor(mean*3);

run("Max...","value=u"); //constraining maximum of 3-fold mean to reduce effect of extreme values during subtraction

//gaussian blurring (step II)

run("Gaussian Blur...", "sigma=100 scaled"); //blurring for subtraction mask

//subtract subtraction mask from image (step III)

selectWindow("IMAGE");

pxrollrad = cellradius/pixelWidth; //rolling ball radius in pixels needed (= predefined cell radius[µm]/pixelsize[µm/px])

run("Subtract Background...", "rolling="+pxrollrad+"");

run("Gaussian Blur...", "sigma=2.5 scaled");//reduces punctate character of grayscale image

imageCalculator("SUBTRACT create", "IMAGE","SUBTRACT");

selectWindow("Result of IMAGE");

run("Duplicate...", "title=AND");//watershed

run("Duplicate...", "title=CHECK");//for checking if maxima exist within selection later

//Apply threshold to get binary image of cell borders (step IV)

selectWindow("Result of IMAGE");

run("Threshold...");

setThreshold(1,256);

setOption("BlackBackground", true);

run("Convert to Mask", "method=Default background=Dark only black");

run("Fill Holes");

//Create watershed line image (step V)

selectWindow("AND");

run("Gaussian Blur...", "sigma=2 scaled");

getVoxelSize(w, h, d, unit);

getStatistics(area, mean, min, max, std);

row = nResults;

setResult("Max ", row, max);

nBins = 256;

getHistogram(values, count, nBins);

//mean gray value of pixels belonging to cells needed (i.e. mean of ONLY non-zero pixel)

Sum = 0;//all counts

CV = 0; //weighed counts (= counts * intensity)

for (i = 1; i<count.length; i++){ //starting with 1 instead of 0. -> 0 intensity values are not considered.

Sum += count[i];

CV += count[i]*i;

}

m = (CV/Sum);

floor(m);

l = floor(2*m); //Maxima need to be at least twice the intensity of cellular mean intensity

run("Find Maxima...", "noise=l output=[Segmented Particles] exclude");//watershedding

//Combine watershed lines and cell frame (step VI)

selectWindow("Result of IMAGE");

imageCalculator("AND create", "Result of IMAGE","AND Segmented");

//select single cells with a spread area > 200 µm (step VII)

run("Analyze Particles...", "size=200.00-Infinity circularity=0.1-1.00 add exclude"); //Cell bodies detected

//Closing windows

selectWindow("AND");

run("Close");

selectWindow("IMAGE");

run("Close");

selectWindow("Result of IMAGE");

run("Close");

selectWindow("AND Segmented");

run("Close");

selectWindow("SUBTRACT");

run("Close");

selectWindow("Result of Result of IMAGE");

run("Close");

//OPTIONAL: Check for nuclei within detected cell borders.

//1/5 of global maximum intensity (corresponding to most intensively stained nucleus) is taken as minimum value to be present in each detected cell frame.

selectWindow("CHECK");

getVoxelSize(w, h, d, unit);

getStatistics(area, mean, min, max, std);

row = nResults;

setResult("Max ", row, max);

maxicheck = floor(max/5); //change for different minimum value

maxtrack = 0; //tracks occasions of cell frames without minimum intensity. Necessary to delete the right frames

roiManager("Multi Measure");

ROInumber = roiManager("count");

for(i = 1; i < ROInumber+1; i++){

cellarea = 0;

cellIntDen = 0;

circularity = 0;

Aratio = 0;

Roundness = 0;

for (a=0; a<nResults(); a++) {

cellarea = getResult("Area"+i+"",a);

circularity = getResult("Circ."+i+"",a);

Aratio = getResult("AR"+i+"",a);

Roundness = getResult("Round"+i+"",a);

Maxvalue = getResult("Max"+i+"",a);

}

if(Maxvalue > maxicheck){

selectWindow("Cell_summaries");

print(f,i+"\t"+cellarea+"\t"+circularity+"\t"+Aratio+"\t"+Roundness+"\t"+Maxvalue);

}

else if (Maxvalue < maxicheck){

maxtrack = maxtrack+1;

roiManager("select", i-maxtrack); //selects the roi without maximum

roiManager("Delete"); //deletes the roi without maximum

}

}

//save results and images (raw brightfield and cellframe overlays)

selectWindow("Cell_summaries");

saveAs("Text", ""+output+"\\"+prefix+".xls");

selectWindow(""+prefix+" C="+y+"");

run("Z Project...", "projection=[Sum Slices]");

roiManager("Show All without labels");

roiManager("Set Color", "red");

roiManager("Set Line Width", 4);

saveAs("Tiff", ""+output+"\\"+prefix+"_OV.tif");

roiManager("Show None");

saveAs("Tiff", ""+output+"\\"+prefix+".tif");

run("Close All");

selectWindow("Cell_summaries");

run("Close");

run("Clear Results");

ROInumber = roiManager("count");

if(ROInumber>0){

roiManager("Delete");

}

}
